# Immunogenicity of a Recombinant MVA Vector Vaccine Expressing the Prefusion RSV F Protein in Balb/c Mice

**DOI:** 10.3390/vaccines14040317

**Published:** 2026-03-31

**Authors:** Jinhui Miao, Min Liu, Qun Zhang, Feixia Gao, Yongshan Zheng, Cheng He

**Affiliations:** 1 Shanghai Institute of Biological Products, Shanghai 200051, China; miaojinhui2026@126.com (J.M.);; 2 State Key Laboratory of Novel Vaccines for Emerging Infectious Diseases, China National Biotec Group Company Limited, Beijing 100024, China

**Keywords:** RSV, MVA, immunogenicity, vaccine, heterologous prime–boost regimen, prefusion F protein

## Abstract

**Background**: Respiratory syncytial virus (RSV) is a leading cause of lower respiratory tract infections and poses a substantial disease burden to infants, older adults, and immunocompromised populations. **Methods**: In this study, a recombinant vaccinia virus (rMVA-RSV preF) was constructed based on the modified vaccinia virus Ankara (MVA) platform by inserting a stabilized prefusion F protein gene of RSV into the MVA genome. The immunogenicity of rMVA-RSV preF and preF protein was evaluated in Balb/c mice under different vaccination regimens. **Results**: A heterologous prime–boost regimen, priming with rMVA-RSV preF and boosting with AS01E-adjuvanted preF protein, elicited robust cellular and humoral immune responses with a Th1 bias. This regimen significantly enhanced immunogenicity compared to homologous vaccination. **Conclusions**: There is a lack of data from further challenge studies to support the efficacy of the rMVA-RSV preF vaccine in terms of protection, but our findings demonstrate a favorable immunogenicity profile of the rMVA-RSV preF vaccine, supporting its further development as a promising RSV vaccine candidate.

## 1. Introduction

Respiratory syncytial virus (RSV) is one of the predominant pathogens causing lower respiratory infections. In 2021, RSV-associated lower respiratory infections resulted in 31,525 deaths worldwide, with an age-standardized mortality rate of approximately 0.5 per 100,000 population [1]. Although public health improvements have led to an overall decline in RSV-related deaths over the past 30 years, the disease burden remains substantial among infants, young children, and the elderly [2]. Current antiviral treatments for RSV have limited efficacy and few approved options. Ribavirin, a broad-spectrum antiviral, is approved for use, but its application is restricted due to side effects [3]. Nirsevimab, an antibody approved in 2023, is currently the most effective therapeutic agent against this virus, widely used for prevention and treatment in pediatric patients [4,5,6]. Consequently, active immunization through vaccination in infants, children, and adults is essential [7,8]. However, RSV vaccine development has faced prolonged challenges, largely due to the historical failure of the formalin-inactivated RSV vaccine (FI-RSV) [9]. RSV can evade host immune responses by skewing immunity toward a Th2-biased profile and elevating IgE levels [10]. It can also cause enhanced respiratory disease (ERD) through pathogenic Th2 memory responses and pulmonary eosinophilic reactions [11]. Inducing RSV-neutralizing antibodies and avoiding the induction of a Th2-biased immune response have become key focuses in vaccine development.

The strong conservation of the respiratory syncytial virus fusion (F) glycoprotein [12] has established it as a primary target for vaccine design and development. The identification of highly potent neutralizing antibodies directed against the prefusion conformation of the F protein (preF) has opened promising avenues for RSV vaccine design [13]. In 2013, the McLellan team first stabilized and engineered the RSV preF antigen using antibody-guided design. Through years of iterative optimization, three major stabilized preF structures have been developed: DS-Cav1, SC-TM, and DS2 [14,15,16]. These stabilized prefusion antigens have significantly advanced RSV vaccine candidates. Notably, the three FDA-approved RSV vaccines—Arexvy (RSVPreF3), Abrysvo (RSVpreF), and mRESVIA (mRNA-1345)—all incorporate a stabilized preF antigen. Each elicits robust neutralizing antibody responses in humans and provides protection at the population level [17]. However, Arexvy and Abrysvo have been associated with cases of Guillain–Barré syndrome following vaccination [18,19]. Additionally, another multivalent vaccine candidate, mRNA-1365 (RSV), encountered safety issues during clinical trials in young children [20]. Beyond these, no RSV vaccines are currently approved for use in young children, with only Abrysvo authorized for administration to pregnant women [21]. This highlights the ongoing limitations in vaccine options.

Modified vaccinia virus Ankara (MVA) was generated by serial passage of chorioallantois vaccinia virus Ankara (CVA). Compared to CVA, MVA has lost approximately 15% of its genome, which leads to a markedly restricted host range and the production of only immature viral particles. Despite this, MVA is capable of expressing early, intermediate, and late viral proteins in most cell types, indicating that viral protein synthesis remains intact. MVA is widely used in developing novel vaccines due to its numerous advantages, including an excellent safety profile, a high capacity for accommodating large exogenous genes, the ability to induce strong antigen-specific immune responses, and inherent adjuvant effects [22,23]. Several vaccines have been developed using the MVA platform, targeting diseases such as HIV, malaria, tuberculosis, and influenza [24]. MVA-based RSV vaccine candidates have advanced through preclinical stages [25,26,27,28] but failed to demonstrate success in clinical trials. These candidates elicited only mild-to-moderate immunogenicity in human subjects and provided limited protection against RSV infection, which has been attributed to insufficient induction of neutralizing antibodies [29]. None of these vaccine candidates employed the newly engineered RSV preF structure as a target antigen, which may explain their failure.

This study developed a candidate MVA vaccine expressing a stable fusion preF protein (rMVA-RSV preF) and evaluated its immunogenicity in Balb/c mice through multiple homologous or heterologous immunization regimens in combination with an RSV preF subunit vaccine. We systematically evaluated post-immunization antigen-specific antibody titers and the secretion of multiple cytokines to preliminarily assess the developmental potential of the candidate vaccine prior to subsequent protective efficacy studies.

## 2. Materials and Methods

### 2.1. Cells and Virus

BHK-21 cells (ATCC CCL-10) were cultured in MEM medium (Gibco, Grand Island, NY, USA, No. 12360-038) containing 10% fetal bovine serum (TransGen Biotech, Beijing, China, No. FS301-02) at 37 °C under 5% CO_2_.

Modified vaccinia virus Ankara (MVA, GenBank: U94848.1) was obtained from ATCC (VR-1508) and stored at −80 °C prior to use. The MVA titer was determined by TCID_50_ assay in BHK-21 cells [30].

### 2.2. Vaccine Design

The rMVA-RSV preF construct was generated via homologous recombination [31]. The DS2 structural design sequence encoding the RSV A2 strain (GenBank accession no: ACO83301.1) fusion glycoprotein (F) was synthesized by GenScript (Nanjing, China). DS2 enables stable expression of the preF structure through modifications including amino acid substitutions, replacement of the p27 sequence with a GS linker, hydrophobic cavity filling, and introduction of disulfide bonds and proline residues [16]. The RSV preF expression cassette, under the control of the vaccinia virus p11 promoter, was cloned into a shuttle plasmid together with the npt selection marker flanked by MVA deletion III homology arms. This yielded the transfer plasmid pMVA-RSV preF; a control plasmid, pMVA-Mock, lacking the RSV preF insert, was constructed in parallel. Subsequently, each plasmid was transfected using X-tremeGENE 9 DNA Transfection Reagent (Roche, Basel, Switzerland, No. 6365779001) into BHK-21 cells that had been pre-infected with wild-type MVA. Recombinant viruses were selected and purified in MEM containing 10% FBS and 600 µg/mL G418 sulfate (Gibco, Grand Island, NY, USA, No. 10131035).

To confirm whether recombinant MVA had successfully incorporated the insert via homologous recombination, specific primers were designed to flank the deletion III locus of MVA. Viral DNA was extracted from BHK-21 cells infected with rMVA or MVA-Mock at 72 h post-infection, using the EasyPure^®^ Viral DNA/RNA Kit (TransGen, No. ER201-01) according to the manufacturer’s protocol. The purified DNA was then amplified by PCR using the primers forward del-F(5’-GATAATAGAACTTACGCAAATATTAGC-3’) and reverse del-R(5’-GCTAAAAGAATAATGGAATTGGGCTC-3’).

### 2.3. Vaccine Characterization

To analyze RSV preF expression, BHK-21 cells infected with rMVA were harvested at the indicated time points. Cells were lysed on ice for 10 min using Pierce™ IP Lysis Buffer (Thermo Fisher, Waltham, MA, USA, No. 87788) containing a protease inhibitor cocktail (Thermo Fisher, No. A32955). Lysates were centrifuged at 10,000 rpm for 10 min at 4 °C, and the supernatant was collected. The supernatant was separated by SDS-PAGE and transferred to a PVDF membrane for Western blot analysis. The membrane was blocked with 5% non-fat dry milk in 1× PBST (pH 7.3) for 2 h at room temperature. Following blocking, the membrane was incubated overnight at 4 °C with a rabbit anti-F primary antibody (SinoBiological, No. 11049-R338) at a dilution of 1:1000. After three washes with PBST, the membrane was incubated for 45 min at room temperature with an HRP-conjugated goat anti-rabbit IgG secondary antibody (Abcam, Cambridge, UK, ab97051). The membrane was washed three times with PBST, and protein bands were visualized using Amersham ECL Prime (Cytiva, Marlborough, MA, USA, RPN2232).

RSV preF protein in the supernatant of rMVA-infected BHK-21 cell lysates was detected using the Human Respiratory Syncytial Virus (RSV) (A2) Prefusion Glycoprotein F/RSV preF ELISA Kit (SinoBiological, Beijing, China, No. KIT11049PR). According to the manufacturer’s instructions, the pre-coated microplate strips (SinoBiological, Beijing, China, No. KIT11049PR) and all reagents were first equilibrated at room temperature for 30 min. After two washes with the provided wash buffer, samples and standards were added to the wells and incubated at room temperature for 1 h. Following three additional washes, the detection antibody working solution was added and incubated for 1 h at room temperature. The plate was washed again three times, after which the substrate solution was added and incubated in the dark at room temperature for 20 min. Finally, the stop solution was added, and the absorbance was measured at 450 nm using a microplate reader (Molecular Devices, San Jose, CA, USA, SpectraMax i3x M5).

MVA was propagated in BHK-21 cells for 72 h at a multiplicity of infection (MOI) of 0.1. Following repeated freeze-thaw cycles and brief ultrasonication, the supernatant was collected. The viral particles were then purified by ultracentrifugation through a 36% sucrose cushion (4 °C, 20,000 rpm, 2 h). The purified virus was resuspended and stored in 10 mmol/L Tris-HCl buffer (pH 9.0).

The DS2 sequence in the RSV preF subunit vaccine is fused with a 6×His tag and expressed in mammalian CHO cells by Shanghai Institute of Biological Products.

### 2.4. Immunization

The protocol for the animal study (Protocol Number: 2024012) was approved by the laboratory animal management committee and the laboratory animal ethics and welfare protection group of the Shanghai Institute of Biological Products.

**Figure 1:**
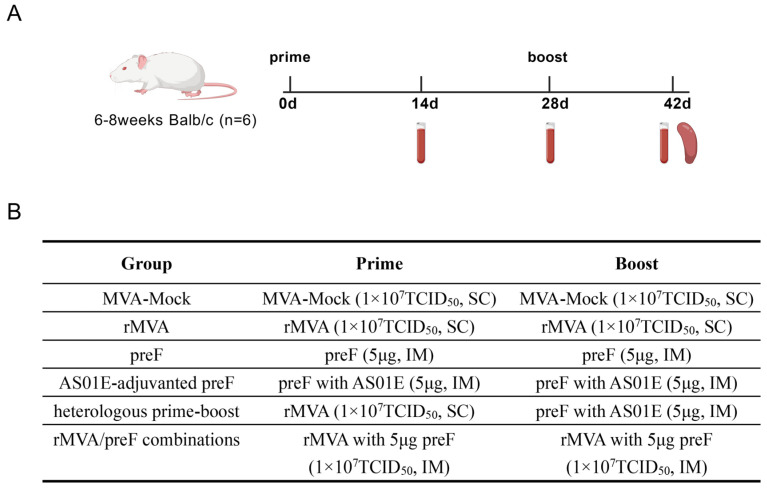
The inoculation, serum, and tissue collection schedule of Balb/c mice: (**A**) A booster vaccination was administered 28 days after the primary immunization. Serum samples were collected on days 14, 28, and 42. Humoral immune responses were analyzed by ELISA and virus-neutralizing antibody assays. On day 42, mice were euthanized, and spleens were collected for the assessment of cellular immune responses using ELISpot and flow cytometry. Created with BioGDP.com (https://biogdp.com/diagram?id=GDPICJV4WH, accessed on 13 January 2026). (**B**) Immunogens and dosages for mice in homologous and heterologous immunization protocols.

Female Balb/c mice were divided into 6 groups (*n* = 6 per group). Within each group, vaccinations were administered via either subcutaneous (SC) or intramuscular (IM) injection at 28-day intervals. Blood samples were collected on days 14, 28, and 42 post-initial immunization. The serum was separated by centrifugation and stored at −80 °C. Following the final blood collection on day 42, mice were euthanized by cervical dislocation. Spleens were harvested for the isolation of lymphocytes, which were subsequently used in cellular immune assays (Figure 1A). The immunogens comprised the rMVA vaccine, preF protein alone, preF protein adjuvanted with AS01E, a combination of rMVA and preF protein, and the control MVA-Mock (Figure 1B).

### 2.5. Antibody Against RSV Prefusion and Post-Fusion F ELISA

Serum levels of antigen-specific binding antibodies were measured using the Mouse Respiratory Syncytial Virus preF Antibody Detection Kit (Vazyme, Nanjing, China, No. DD3910) and postF Antibody Detection Kit (Vazyme, No. DD3911). According to the manufacturer’s instructions, pre-coated plates were first equilibrated to room temperature along with all reagents. Mouse serum samples were subjected to a 3-fold serial dilution before being added to the wells and incubated at 37 °C for 1 h. After four washes with the provided wash buffer, an enzyme-conjugated detection reagent was added and incubated at 37 °C for 1 h. The plates were again washed four times, followed by the addition of substrate solution and incubation in the dark at 37 °C for 15 min. The reaction was stopped with a stop solution. Absorbance was read at 450 nm and 630 nm using a microplate reader (Molecular Devices, San Jose, CA, USA, SpectraMax i3x M5). The optical density (OD) for each well was calculated as OD_450_-OD_630_. The cutoff value was defined as 0.09 plus the mean OD of the negative control wells (with values < 0.05 set to 0.05). The endpoint titer for each sample was reported as the highest dilution factor that yielded an OD above the cutoff.

### 2.6. Serum RSV preF-Specific IgG Subclass ELISA

A 96-well ELISA plate (Corning, Corning, NY, USA, No. 9018) was coated with RSV preF protein (100 ng/well) diluted in CBA buffer (pH 9.6) overnight at 4 °C. After three washes with PBST, the plate was blocked with 5% non-fat dry milk in PBST at 37 °C for 2 h. Following another three washes, mouse serum samples, serially diluted 3-fold, were added to the wells and incubated at 37 °C for 2 h. The plate was washed three times and then incubated with HRP-conjugated goat anti-mouse IgG1 or IgG2a secondary antibody (SouthernBiotech, Beijing, China, 5300-05) at 37 °C for 1 h. After a final wash step, TMB substrate (InnoReagents, Deqing, China, TMB-S-001) was added, and the plate was incubated in the dark at 37 °C for 15 min. The reaction was stopped by adding 2M H_2_SO_4_. Absorbance at 450 nm was measured using a microplate reader (Molecular Devices, San Jose, CA, USA, SpectraMax i3x M5). The cutoff value was defined as the mean OD of negative control wells (serum from non-immunized mice) + 2 × SD. The endpoint titer was reported as the highest serum dilution that yielded an OD exceeding the cutoff.

### 2.7. Virus Neutralization Assay

Diluted test samples and positive control serum were added to a 96-well plate (Gibco, Grand Island, NY, USA, No. 701001). Then, 500 plaque-forming units (PFUs) of RSV-A2 (ATCC VR-1540) were added to each well, and the plate was incubated at 37 °C with 5% CO_2_ for 1 h. The virus-serum mixture was subsequently transferred to Hep-2 cell monolayers (ATCC CCL-23) that had been cultured at 37 °C with 5% CO_2_ overnight. After incubation at 37 °C with 5% CO_2_ for 2 h, the supernatant was removed. Fresh culture medium was added, and the cells were cultured for another 22 h. Following the removal of the supernatant, the cells were fixed with 4% paraformaldehyde (Beyotime, Shanghai, China, No. P0099) and then incubated with a fluorescently labeled detection antibody (Vazyme, No. DD1606). After staining, the plates were read using an immunofluorescence plate reader (CTL, Shaker Heights, OH, USA, S6 Universal M2). The neutralizing antibody titer was defined as the highest serum dilution that neutralized 50% of the viral infection.

### 2.8. Enzyme-Linked Immunospot Assay

On day 42, mice were euthanized, and spleens were harvested. A single-cell suspension was prepared from splenic tissue using a dissociation buffer (Dakewe, Shenzhen, China, No. 7211011). Subsequent steps followed the manufacturer’s instructions for the mouse IFN-γ ELISpot kit (Mabtech, Nacka Strand, Sweden, No. 3321-4HST-10). Briefly, 5 × 10^5^ cells were seeded per well and stimulated with the RSV A2 F protein peptide pool (GenScript, Nanjing, China, SIBP-RA). After incubation at 37 °C with 5% CO_2_ for 48 h, spot-forming units (SFUs) were quantified using an automated reader (CTL, S6 Universal M2). The background value, derived from unstimulated control wells, was subtracted from the stimulated well counts.

### 2.9. Cytokine Analysis

Cells were seeded at 5 × 10^6^ cells per well in a 48-well culture plate (Corning, Corning, NY, USA, No. 3548) and stimulated with the RSV A2 F protein peptide pool (GenScript, Nanjing, China, SIBP-RA). After incubation at 37 °C with 5% CO_2_ for 72 h, cell culture supernatants were collected. Cytokine concentrations in the supernatants were measured using the LEGENDplex™ MU Th Cytokine Panel (12-plex) kit (BioLegend, San Diego, CA, USA, No. 741044) following the manufacturer’s protocol. The processed samples were acquired on a flow cytometer (Agilent NovoCyte, Santa Clara, CA, USA, Advanteon), and data were analyzed with the corresponding LEGENDplex™ software version 8.0 (https://legendplex.qognit.com/workflow, accessed on 3 December 2025).

### 2.10. Statistical Analysis

Statistical analysis was performed using GraphPad Prism 9.0 software. For comparisons across multiple groups under a single factor, one-way ANOVA was performed, followed by Tukey’s multiple-comparison test. *p* values are denoted as follows: * *p* < 0.05, ** *p* < 0.01, *** *p* < 0.001, **** *p* < 0.0001.

## 3. Results

### 3.1. Construction and Characterization of the Recombinant MVA Vaccine Expressing RSV preF

**Figure 2:**
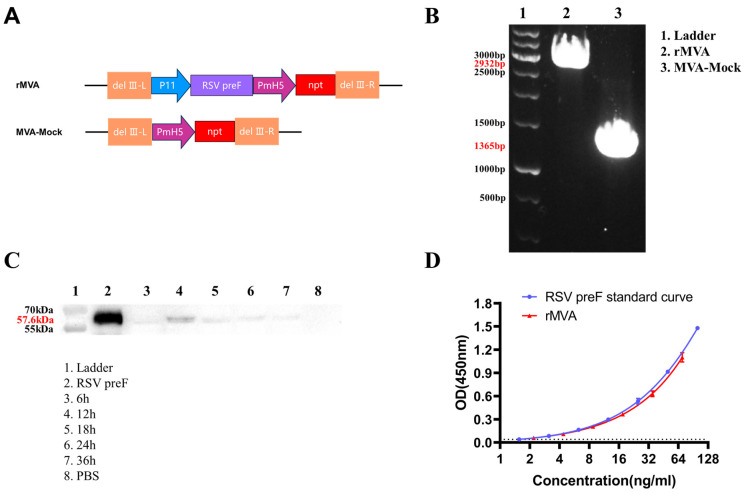
Construction and characterization of recombinant MVA vaccine: (**A**) The gene sequence encoding the stabilized prefusion F protein of RSV A (RSV preF) was inserted into the deletion III site of the MVA genome. The control recombinant MVA carrying only the neomycin phosphotransferase (npt) selectable marker was generated in parallel. (**B**) PCR with primers flanking the insertion site confirmed successful recombination. The amplified fragment from the RSV preF-containing construct was 2932 bp, while that from the npt-only control was 1365 bp. (**C**) BHK-21 cells were infected with rMVA at an MOI of 0.5. Lysates collected at the indicated time points were analyzed by Western blot under reducing and denaturing conditions. (**D**) An RSV preF-specific ELISA was used to evaluate the antigenic integrity and binding capacity of the F protein expressed in rMVA-infected BHK-21 cells, using conformation-specific monoclonal antibodies. Dashed lines denote the lower limit of detection.

The gene sequence encoding the stabilized prefusion F protein of RSV A (RSV preF) was inserted into the deletion III site of the MVA genome through homologous recombination, under the control of the vaccinia virus P11 promoter. The npt selection marker was co-inserted during this process (Figure 2A). PCR with primers flanking the MVA insertion site confirmed the isolation of pure recombinant virus (rMVA) and the control virus (MVA-Mock), yielding specific amplicons of 2932 bp and 1365 bp, respectively. No wild-type MVA contamination was detected, as indicated by the absence of the 500 bp band (Figure 2B). Western blot analysis of lysates from rMVA-infected cells confirmed expression of the preF antigen, with a distinct band observed at ~57.6 kDa (Figure 2C). A time-course study showed that the preF-specific signal was detectable as early as 6 h post-infection, peaked at 12 h, and subsequently declined (Figure 2C). Furthermore, ELISA using conformation-specific antibodies demonstrated that the F protein expressed by rMVA retained antigenic features characteristic of the prefusion state, showing reactivity comparable to that of a reference preF standard included in the kit, consistent with the designed stabilization strategy (Figure 2D).

### 3.2. Recombinant Vaccines Induce Antigen-Specific Humoral Responses in Mice

**Figure 3:**
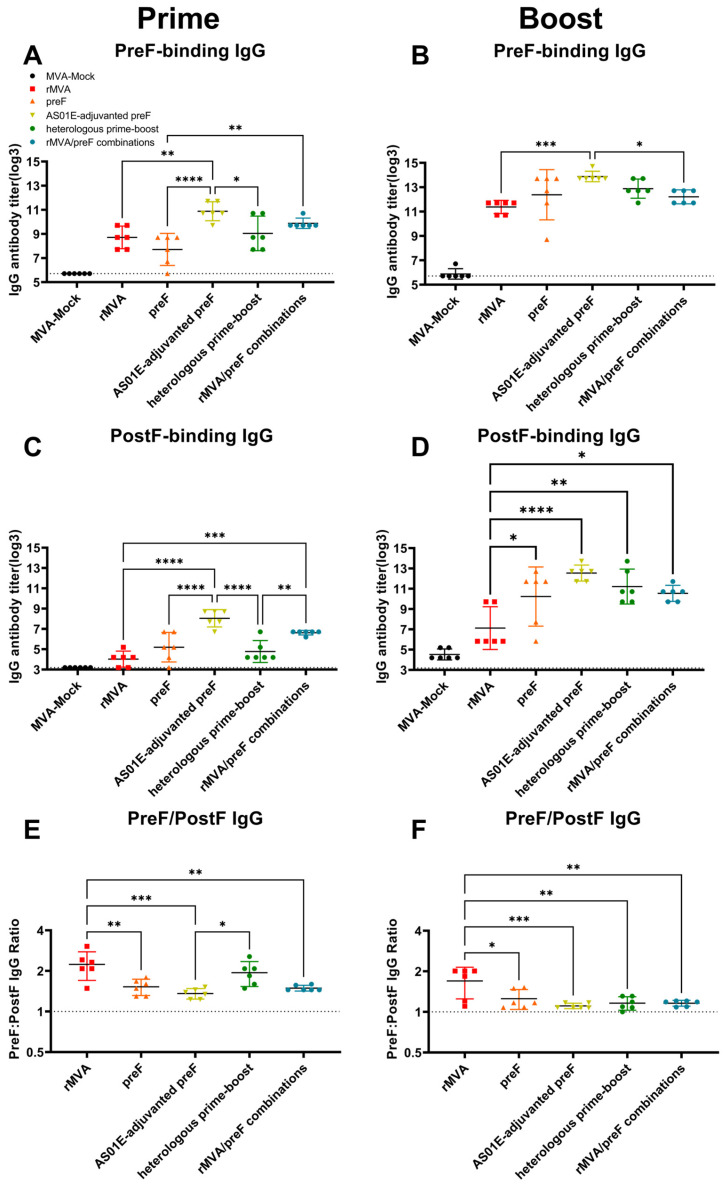
Vaccine-induced antigen-specific humoral immunity: (**A**,**B**) Titers of RSV preF-specific binding antibodies. (**C**,**D**) Titers of RSV postF-specific binding antibodies. (**E**,**F**) Ratio of antigen-specific binding antibody titers. Horizontal lines indicate the group means with 95% confidence intervals. Dashed lines denote the lower limit of detection (**A**–**D**), preF/postF equilibrium (**E**,**F**). * *p* < 0.05, ** *p* < 0.01, *** *p* < 0.001, **** *p* < 0.0001. Comparisons between the MVA-Mock group and other groups were not included in the primary statistical analysis.

To assess the RSV-specific humoral immunity elicited by the recombinant vector vaccine, serum samples collected after primary and booster immunizations were analyzed for antibody responses. Sera from the MVA-Mock control group remained below the seropositivity threshold or showed only minimal background reactivity near the assay’s limit of detection throughout the study. After primary immunization, sera from all experimental groups contained detectable levels of IgG antibodies binding to RSV preF. The preF protein formulated with the AS01E adjuvant elicited a markedly stronger antibody response. The responses induced by rMVA alone and the rMVA/preF combination were comparable in magnitude, while the adjuvant-free preF protein elicited weaker antibody titers (Figure 3A). Following booster immunization, a marked increase in serum antibody titers was observed across all experimental groups. Statistical analysis indicated that the AS01E-adjuvanted preF group differed significantly from both the rMVA group and the rMVA/preF combination group, whereas no statistically significant difference was detected relative to the heterologous prime–boost group (Figure 3B). Consistent with the trend in RSV preF-binding antibody titers, the AS01E-adjuvanted preF group also showed the highest titers against RSV postF. Notably, the rMVA group induced lower postF-binding antibody titers compared to the preF group, although this difference was not statistically significant (Figure 3C). After the booster immunization, all experimental groups except the rMVA group demonstrated increased postF-binding antibody responses, which were significantly higher than those in the rMVA group (Figure 3D). Throughout the immunization period, the preF/postF antibody titer ratio remained above 1 in all groups, indicating a consistent bias of the humoral response toward the stabilized prefusion conformation of the F protein. Following the booster with AS01E-adjuvanted preF, the ratio in the heterologous prime–boost group declined significantly. In contrast, the rMVA group maintained a higher ratio, which differed significantly from the other experimental groups (Figure 3E,F).

**Figure 4:**
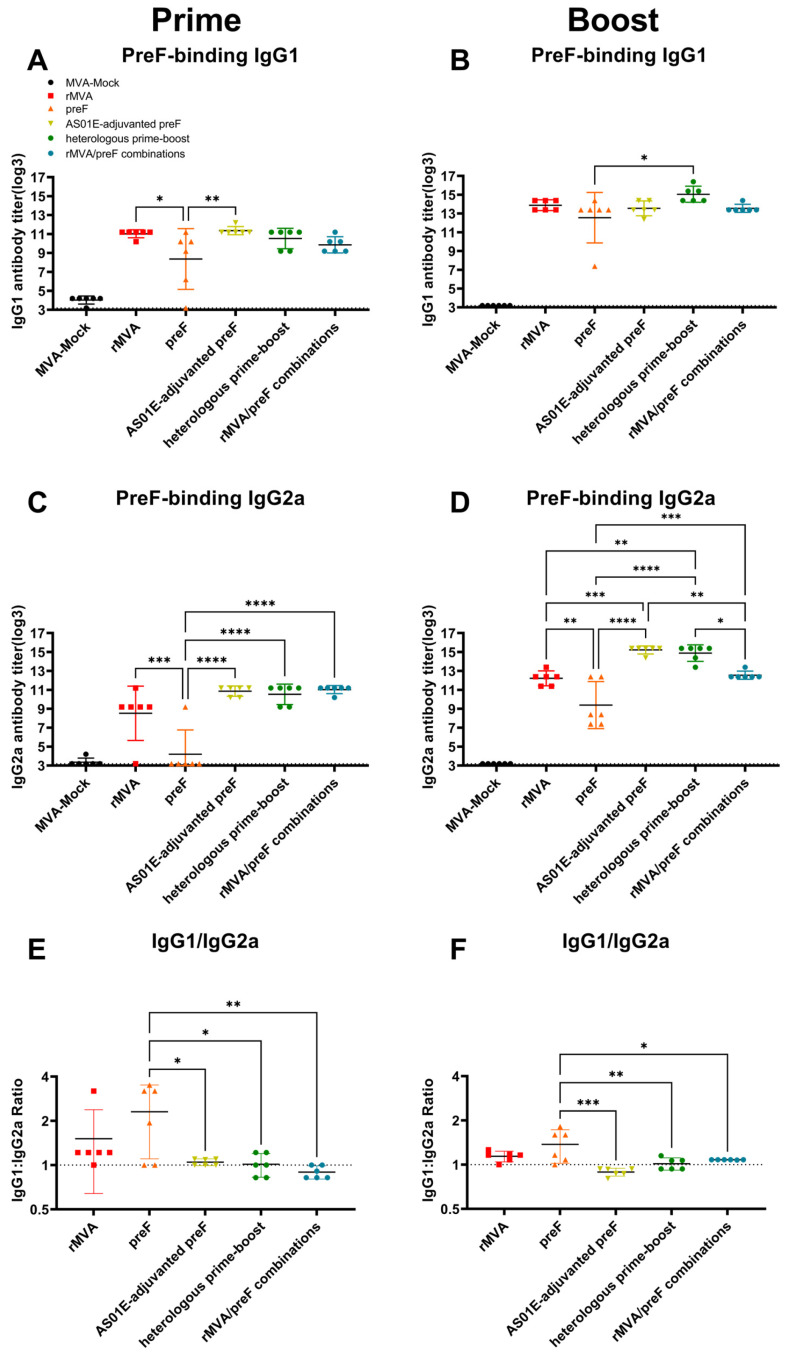
Vaccine-induced antigen-specific humoral immunity (IgG1 and IgG2a): (**A**,**B**) Titers of preF-specific IgG1 antibodies. (**C**,**D**) Titers of preF-specific IgG2a antibodies. (**E**,**F**) IgG1/IgG2a ratio. Horizontal lines indicate the group means with 95% confidence intervals. Dashed lines denote the lower limit of detection (**A**–**D**) and IgG1/IgG2a equilibrium (**E**,**F**). * *p* < 0.05, ** *p* < 0.01, *** *p* < 0.001, **** *p* < 0.0001. Comparisons between the MVA-Mock group and other groups were not included in the primary statistical analysis.

This study assessed the Th1/Th2 polarization of the immune response by isotyping preF-specific antibodies following immunization. All experimental groups except the preF group induced high titers of specific IgG1 antibodies after immunization, with no statistically significant differences observed among these groups. However, the preF group showed a statistically significant difference compared to the group with the highest titer (Figure 4A,B). The pattern of IgG2a antibody titers after primary immunization was similar to that of IgG1, with the low-titer preF group differing significantly from the other groups (Figure 4C). After boosting, the two groups receiving AS01E-adjuvanted preF protein (the AS01E-adjuvanted preF group and the heterologous prime–boost group) elicited the highest IgG2a titers, followed by the rMVA group and the rMVA/preF combination group (Figure 4D). The preF group exhibited a higher IgG1/IgG2a ratio compared to the other experimental groups, whose ratios were closer to 1, indicating a relatively balanced Th1/Th2 response in all groups except the preF group (Figure 4E,F). Following booster immunization, multiple experimental groups successfully induced high levels of specific antibodies, along with elevated preF-binding antibodies and a Th1-biased immune response.

**Figure 5:**
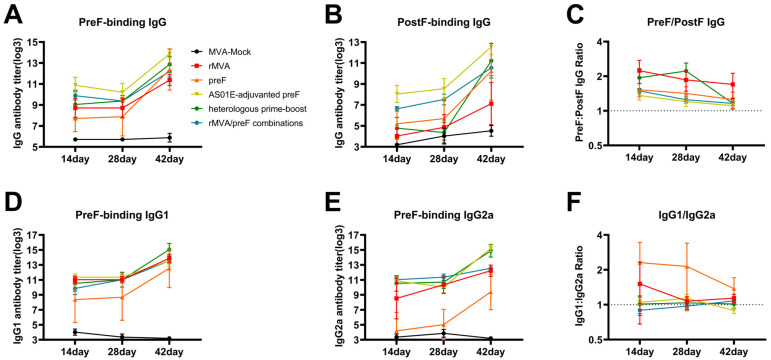
Vaccine-induced antigen-specific humoral immune changes: (**A**–**F**) Serum samples from 6 mice per group were collected on days 14, 28, and 42 post-immunization and assayed by ELISA to measure antibody titers. Parameters assessed included reactivity against RSV preF (**A**) and postF (**B**) antigens and the preF/postF binding ratio (**C**), as well as titers of preF-specific IgG1 (**D**) and IgG2a (**E**) antibodies and the corresponding IgG1/IgG2a ratio (**F**). Data are presented as the mean antibody titer ± standard deviation for each group (*n* = 6). The dashed lines indicate a ratio of 1, representing the preF/postF equilibrium (**C**) and the IgG1/IgG2a equilibrium (**F**).

Antibody titers against RSV preF remained stable or declined gradually during the 28 days following primary immunization. After the booster dose, all groups showed a substantial increase in titers, with the greatest rise observed in the preF group (Figure 5A). Titers against RSV postF increased modestly by day 28 in all groups except the heterologous prime–boost group. Following the booster, postF-binding titers rose markedly across all groups, with the heterologous prime–boost group exhibiting the most pronounced increase (Figure 5B). The preF/postF titer ratio remained above 1 in all groups but decreased slightly after the booster immunization (Figure 5C). Consistent with the trend for total preF-binding antibodies, titers of preF-specific IgG1 and IgG2a subclasses also rose significantly after the booster (Figure 5D,E). Conversely, the IgG1/IgG2a ratio gradually declined over time, approaching a value of 1 (Figure 5F). Different immunization regimens significantly enhanced antigen-specific antibody levels after booster vaccination and maintained a favorable Th1/Th2 immune balance.

**Figure 6:**
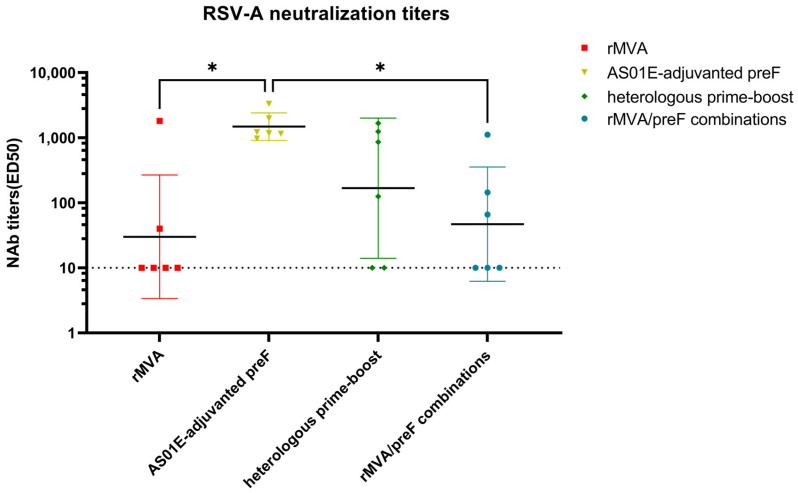
RSV-A neutralizing activity in day 42 serum samples. The horizontal lines denote the geometric mean with 95% confidence intervals for each group; the dashed line indicates the lower limit of detection. * *p* < 0.05.

The titer of RSV-neutralizing antibodies serves as a key correlate of protective immunity induced by vaccination [32]. The preF protein adjuvanted with AS01E elicited high neutralizing antibody titers, whereas the other groups showed low to undetectable levels (Figure 6). The AS01E-adjuvanted preF group differed significantly from both the rMVA group and the rMVA/preF combination group. Although the heterologous prime–boost regimen induced lower neutralizing antibody titers, no statistically significant difference was observed between this group and the AS01E-adjuvanted preF group (Figure 6). This result indicates that recombinant MVA vaccines play only a minimal role in inducing neutralizing antibodies, whereas recombinant protein vaccines are critical for generating high levels of neutralizing antibodies. Previous results showed that the Mock group had extremely low levels of specific binding antibodies. Given that the Mock control group was already negative for binding antibodies, the likelihood of it producing specific neutralizing activity was theoretically negligible; therefore, neutralizing antibody titers were not measured.

### 3.3. Recombinant Vaccines Induce Cellular Immune Responses in Mice

**Figure 7:**
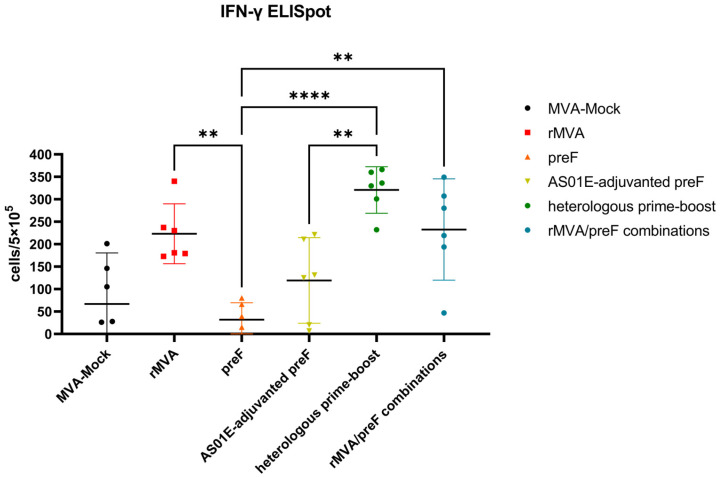
Cellular immune responses following immunization in mice. Splenocytes from immunized mice were stimulated with a peptide pool spanning the RSV A F protein. IFN-γ-expressing cells were quantified by ELISpot, with results presented as spot-forming units per 5 × 10^5^ splenocytes. Horizontal lines indicate the group means with 95% confidence intervals. ** *p* < 0.01, **** *p* < 0.0001. Comparisons between the MVA-Mock group and other groups were not included in the primary statistical analysis. Data points below the assay detection limit are not depicted in the figure.

Cellular immune responses induced by vaccination were assessed via IFN-γ ELISpot. The MVA-Mock control group exhibited background IFN-γ secretion similar to that of the preF group (Figure 7). Although the AS01E-adjuvanted preF group showed slightly higher IFN-γ responses compared to both the MVA-Mock and preF groups, no statistically significant differences were observed among these three groups. In contrast, the rMVA, heterologous prime–boost, and rMVA/preF combination groups all elicited strong IFN-γ production. While no statistically significant differences were detected between them, the heterologous prime–boost group displayed the highest mean level of IFN-γ secretion (Figure 7).

**Figure 8:**
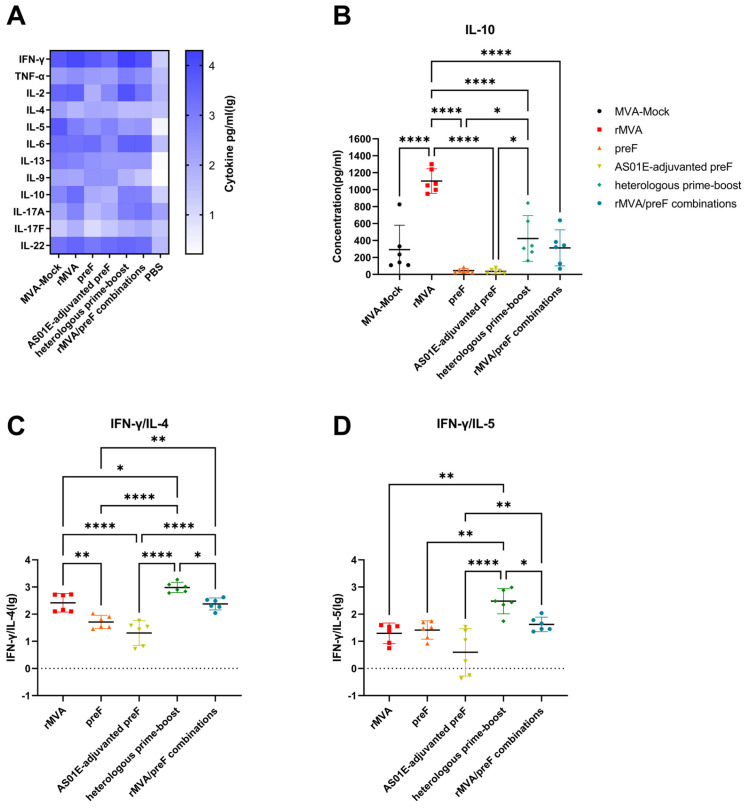
Cytokine secretion profile elicited by vaccination in mice: (**A**) Mouse splenocytes were stimulated for 48 h with the RSV F protein peptide pool. Supernatants were collected and analyzed by bead-based multiplex immunoassay (LEGENDplex™) to quantify secreted cytokines. (**B**) IL-10 concentration. (**C**) IFN-γ/IL-4 ratio. (**D**) IFN-γ/IL-5 ratio. The horizontal lines indicate the group means with 95% confidence intervals. The dashed lines denote immune equilibrium (**C**,**D**). * *p* < 0.05, ** *p* < 0.01, **** *p* < 0.0001.

To further characterize the vaccine-induced cellular immunity, cytokine secretion profiles were analyzed using a multiplex assay. The cytokine levels detected in splenocyte supernatants correlated with the ELISpot findings (Figure 8A). Immunization with MVA-based vaccines triggered the secretion of a broad spectrum of T helper (Th) cell-associated cytokines. Among the Th1-type cytokines(IFN-γ, TNF-α, and IL-2), a similar pattern of differences was observed across the groups. The heterologous prime–boost group secreted significantly higher levels of these cytokines compared to other groups. In contrast, the preF group and the AS01E-adjuvanted preF group showed secretion levels similar to those of the non-specific stimulation control (Supplementary Figure S1A–C). Among Th2-associated cytokines (IL-4, IL-5, and IL-13), the background secretion level in the nonspecifically stimulated MVA-Mock control group was significantly higher than in all other groups. The preF group showed elevated IL-4 secretion compared to the other experimental groups, whereas IL-5 and IL-13 levels were similar across the remaining experimental groups (Supplementary Figure S1D–F). Secretion of the pro-inflammatory cytokine IL-9 was comparable among all groups (Supplementary Figure S1G). Notably, IL-10 secretion was significantly higher in the rMVA group than in the other groups. Furthermore, the heterologous prime–boost group secreted more IL-10 than both the preF group and the AS01E-adjuvanted preF group (Figure 8B). Finally, among Th17-associated cytokines (IL-17A, IL-17F, IL-6, and IL-22), the rMVA, heterologous prime–boost, and rMVA/preF combination groups showed higher secretion levels compared to the remaining groups, although considerable animal-to-animal variability was noted within these high-responding groups (Supplementary Figure S1I–L). The heterologous prime–boost group displayed an elevated IFN-γ/IL-4 ratio, indicative of an increased Th1-skewed immune bias. The ratios in the rMVA and rMVA/preF combination groups were also elevated relative to those in the preF and AS01E-adjuvanted preF groups (Figure 8C). Regarding the IFN-γ/IL-5 ratio, which reflects the balance of Th1 immunity relative to eosinophil-associated responses, the heterologous prime–boost group exhibited a higher ratio than the other groups, whereas the AS01E-adjuvanted preF group showed a lower ratio (Figure 8D). The recombinant MVA vaccine utilizes its unique properties to induce robust cellular immunity along with adjuvant effects while also eliciting a Th1-biased immune response.

## 4. Discussion

The subunit vaccine currently on the market has proven that the RSV preF protein can induce robust antibody responses in mice [33], while the recombinant MVA-based RSV vaccine demonstrates a strong capacity to elicit T-cell responses [28]; these distinct outcomes arise from their different mechanisms of action. Due to its ability to induce high levels of neutralizing antibodies and its high degree of conservation across RSV subtypes, RSV preF has become the most commonly used antigen in current RSV vaccine development [34]. However, previously reported MVA vaccines did not utilize RSV preF as the immunizing antigen. We successfully developed a recombinant MVA vaccine using the stabilized prefusion F protein as the antigen. By employing both homologous and heterologous immunization regimens—specifically, primary immunization with rMVA followed by a booster with AS01E-adjuvanted preF protein—we evaluated the immunogenicity of this candidate vaccine.

Analysis of humoral immunity showed that both vaccine platforms elicited antibody responses. However, two doses of AS01E-adjuvanted preF protein induced stronger immunogenicity than two doses of rMVA. Notably, combining rMVA with preF protein did not enhance the overall humoral response beyond that of the individual components. It should be noted that these three groups differed in administration routes and antigen doses, limiting direct comparability. Previous studies have reported that heterologous prime–boost regimens using MVA often elicit superior humoral responses compared with homologous MVA vaccination [35,36,37,38]. Consistent with this, in our study, the heterologous prime–boost group and AS01E-adjuvanted preF group showed statistically comparable humoral immunity at Day 42 (*p* > 0.05), indicating that the heterologous strategy effectively enhances the antibody response induced by rMVA priming. The correlation between anti-postF and anti-preF antibody titers is attributable to the overlapping antigenic sites shared by both conformations of the F protein [39]. Nevertheless, the higher preF/postF ratio observed in the rMVA group suggests that the antibodies induced by the recombinant MVA vaccine are preferentially directed against epitopes specific to the prefusion conformation. This bias toward the prefusion state was evident both after primary immunization and following the booster dose. In another study using an Ad26 adenovirus-vectored vaccine expressing stabilized prefusion F protein and a subunit RSV vaccine, mice immunized with the preF protein showed a higher preF/postF antibody ratio [40]. This difference may be related to the distinct properties of the viral vector and the immunization protocol. Given the established link between prefusion-specific antibodies and the potential risk of antibody-dependent enhancement (ADE) in RSV infection [41,42], our findings suggest that recombinant MVA-vectored RSV vaccines might present a lower risk of ADE compared to subunit or adenovirus-vectored vaccines. Although the rMVA group induced lower overall humoral immunogenicity than other experimental groups, it generated IgG1 antibody titers comparable to those of the other groups. After two immunizations, both the rMVA and rMVA/preF combination groups exhibited lower IgG2a antibody titers than the AS01E-adjuvanted preF group and the heterologous prime–boost group. This indicates that the IgG response elicited by rMVA is predominantly of the IgG1 subclass. Furthermore, the preF group exhibited lower IgG2a titers and a higher IgG1/IgG2a ratio compared to the other groups, indicating that the adjuvant-free preF protein induced a predominantly IgG1-driven, Th2-skewed response. In contrast, the remaining groups showed a tendency toward a more balanced Th1/Th2 immune profile. Consistent with this, other studies on preF subunit vaccines have also reported that adjuvants significantly modulate the IgG1/IgG2a balance in mice, thereby influencing Th1/Th2 polarization [43,44]. Although avoiding Th2 bias is a major focus in RSV vaccine design [10,11], the licensed subunit vaccine Abrysvo (RSVpreF) employs an adjuvant-free formulation—a decision informed by comprehensive safety and immunogenicity assessments [45]. Its bivalent design, incorporating preF antigens from both RSV subtypes, may contribute to a favorable Th1/Th2 balance. In contrast, the monovalent vaccine Arexvy (RSVPreF3) utilizes the AS01 adjuvant system [33]. It should be noted that the IgG1/IgG2a ratio serves only as an indirect indicator of Th1/Th2 bias; direct assessment of cytokine profiles provides more definitive evidence. Neutralizing antibody data confirm that AS01-adjuvanted preF protein is particularly effective in eliciting potent humoral immunity. Other reports suggest that recombinant viral-vectored vaccines often require higher antigen doses or optimized regimens to achieve neutralizing antibody levels comparable to those induced by adjuvanted protein vaccines [33,40,46,47].

In this study, rMVA primarily elicited cellular immune responses in mice. The rMVA-immunized group produced significantly higher levels of IFN-γ compared to the preF-immunized group, as confirmed by both ELISpot and intracellular cytokine staining. Notably, the heterologous prime–boost group mounted a stronger cellular response than the rMVA group, with IFN-γ secretion substantially exceeding that of other experimental groups. Other Th1-associated cytokines (TNF-α, IL-2) showed secretion patterns similar to IFN-γ, while rMVA also reduced IL-4 production. Furthermore, the MVA-Mock control group non-specifically stimulated Th1 and Th2-associated cytokine secretion, with Th2-associated cytokine levels being particularly higher than in the experimental groups. The elevated IL-10 secretion observed in the rMVA group may be associated with the relatively lower IgG antibody titers, particularly IgG2a, following homologous rMVA boosting. MVA vaccines are known to exert non-specific immunomodulatory effects [48] and can induce immune tolerance in trained immunity models in vitro [49]. However, multiple studies have highlighted a critical immunoregulatory role for IL-10 during RSV infection in mice [50,51,52], suggesting that the elevated IL-10 observed in the rMVA group may contribute to protective immunity against RSV. Compared to subunit vaccines, rMVA vaccination also triggered the secretion of Th17-associated cytokines (IL-17A, IL-17F, IL-6, and IL-22), although considerable variability within these groups was noted. The biological relevance and cause of this high variability remain unclear. Owing to higher IFN-γ and lower IL-4 production, rMVA promoted a Th1-skewed immune response in mice, an effect that was increased in the heterologous prime–boost group. This indicates that an AS01E-adjuvanted preF protein booster enhances polarization toward a Th1-biased profile. No significant differences in antigen-specific immunity were detected between the rMVA group and the rMVA/preF combination group, suggesting that co-administration of the recombinant viral vector and protein antigen does not provide an immunologic advantage under these conditions. Collectively, the heterologous prime–boost regimen elicited high levels of binding antibodies comparable to those induced by adjuvanted subunit vaccines while also generating a robust Th1-skewed cellular immune response.

Our results indicate that recombinant subunit vaccines are effective as boosters in enhancing immunogenicity; however, safety concerns have been reported regarding currently available recombinant protein vaccines. In future experiments, we will focus on the safety profile of recombinant subunit vaccines and conduct a comprehensive assessment of the feasibility of the heterologous prime–boost regimen.

An international standard serum for neutralization assays was not included, preventing calibration of our titers to international units. However, the rigorous inclusion of positive and negative controls in every assay run ensures the internal consistency and reliability of our findings. Future studies should incorporate such standards to enable cross-study comparisons.

Another limitation of this study is the absence of challenge experiments. The neutralizing antibody data for rMVA were poor, and the cytokine levels in the cellular immune response only reflected the characteristics of the recombinant MVA vaccine. Due to the lack of protective studies, it is not possible to determine the efficacy of rMVA in protecting against RSV infection. Given that hRSV replicates inefficiently in conventional Balb/c mice, findings from such experiments may not reliably reflect the protective efficacy of candidate vaccines [53,54]. The present study focuses primarily on evaluating the immunogenicity of the candidate vaccines, and future studies will employ animal models such as cotton rats to enable a more comprehensive assessment.

## 5. Conclusions

Our study shows that the recombinant MVA-vectored RSV vaccine candidate elicits both cellular and humoral immune responses specific to RSV in Balb/c mice, with the induced antibodies primarily targeting conformation-dependent epitopes on the prefusion F protein. While a heterologous prime–boost regimen significantly improved immunogenicity, further evaluation of immunogenicity and protective efficacy in additional animal models is warranted to fully assess this vaccine strategy.

## Data Availability

The data generated in the present study may be requested from the corresponding author.

## Supplementary Materials

The following supporting information can be downloaded at: https://www.mdpi.com/article/10.3390/vaccines14040317/s1, Supplementary Figure S1. Cytokine secretion profiles and intergroup differences following vaccination in mice. Mouse splenocytes were stimulated for 48h with an RSV A F protein peptide pool. Culture supernatants were collected and analyzed by LEGENDplex™ bead-based immunoassay for the following cytokines: IFN-γ (A), TNF-α (B), IL-2 (C), IL-4 (D), IL-5 (E), IL-13 (F), IL-9 (G), IL-10 (H), IL-17A (I), IL-17F (J), IL-6 (K), and IL-22 (L). Horizontal lines represent the group mean with 95% confidence intervals. * *p* < 0.05, ** *p* < 0.01, *** *p* < 0.001, **** *p* < 0.0001.

## References

[1] Chen Z.W., Zhang Q., Li J.R., Xie N.H., Zheng Q.M., Lai Y.Z., Zhang X.Y. (2025). Contribution of Respiratory Syncytial Virus to Burden of Lower Respiratory Tract Infections: A Global Analysis of 204 Countries and Territories, 1990–2021. Trop. Med. Infect. Dis..

[2] Du Y.X., Yan R., Wu X.Y., Zhang X.B., Chen C., Jiang D.X., Yang M.Y., Cao K.X., Chen M.S., You Y. (2023). Global Burden and Trends of Respiratory Syncytial Virus Infection across Different Age Groups from 1990 to 2019: A Systematic Analysis of the Global Burden of Disease 2019 Study. Int. J. Infect. Dis..

[3] Avery L., Hoffmann C., Whalen K.M. (2020). The Use of Aerosolized Ribavirin in Respiratory Syncytial Virus Lower Respiratory Tract Infections in Adult Immunocompromised Patients: A Systematic Review. Hosp. Pharm..

[4] Hammitt L.L., Dagan R., Yuan Y., Cots M.B., Bosheva M., Madhi S.A., Muller W.J., Zar H.J., Brooks D., Grenham A. (2022). Nirsevimab for Prevention of Rsv in Healthy Late-Preterm and Term Infants. N. Engl. J. Med..

[5] Jones J.M., Fleming-Dutra K.E., Prill M.M., Roper L.E., Brooks O., Sanchez P.J., Kotton C.N., Mahon B.E., Meyer S., Long S.S. (2023). Use of Nirsevimab for the Prevention of Respiratory Syncytial Virus Disease among Infants and Young Children: Recommendations of the Advisory Committee on Immunization Practices-United States, 2023. MMWR-Morb. Mortal. Wkly. Rep..

[6] Sumsuzzman D.M., Wang Z., Langley J.M., Moghadas S.M. (2025). Real-World Effectiveness of Nirsevimab against Respiratory Syncytial Virus Disease in Infants: A Systematic Review and Meta-Analysis. Lancet Child Adolesc. Health.

[7] Gatt D., Martin I., AlFouzan R., Moraes T.J. (2023). Prevention and Treatment Strategies for Respiratory Syncytial Virus (Rsv). Pathogens.

[8] Debbag R., Rudin D., Ceddia F., Watkins J. (2025). The Impact of Vaccination on COVID-19, Influenza, and Respiratory Syncytial Virus-Related Outcomes: A Narrative Review. Infect. Dis. Ther..

[9] Kim H.W., Canchola J.G., Brandt C.D., Pyles G., Chanock R.M., Jensen K., Parrott R.H. (1969). Respiratory Syncytial Virus Disease in Infants Despite Prior Administration of Antigenic Inactivated Vaccine. Am. J. Epidemiol..

[10] Becker Y. (2006). Respiratory Syncytial Virus (Rsv) Evades the Human Adaptive Immune System by Skewing the Th1/Th2 Cytokine Balance toward Increased Levels of Th2 Cytokines and Ige, Markers of Allergy-a Review. Virus Genes.

[11] Acosta P.L., Caballero M.T., Polack F.P. (2016). Brief History and Characterization of Enhanced Respiratory Syncytial Virus Disease. Clin. Vaccine Immunol..

[12] Collins P.L., Huang Y.T., Wertz G.W. (1984). Nucleotide-Sequence of the Gene Encoding the Fusion (F) Glycoprotein of Human Respiratory Syncytial Virus. Proc. Natl. Acad. Sci. USA.

[13] Magro M., Mas V., Chappell K., Vázquez M., Cano O., Luque D., Terrón M.C., Melero J.A., Palomo C. (2012). Neutralizing Antibodies against the Preactive Form of Respiratory Syncytial Virus Fusion Protein Offer Unique Possibilities for Clinical Intervention. Proc. Natl. Acad. Sci. USA.

[14] McLellan J.S., Chen M., Joyce M.G., Sastry M., Stewart-Jones G.B.E., Yang Y.P., Zhang B.S., Chen L., Srivatsan S., Zheng A.Q. (2013). Structure-Based Design of a Fusion Glycoprotein Vaccine for Respiratory Syncytial Virus. Science.

[15] Krarup A., Truan D., Furmanova-Hollenstein P., Bogaert L., Bouchier P., Bisschop I.J.M., Widjojoatmodjo M.N., Zahn R., Schuitemaker H., McLellan J.S. (2015). A Highly Stable Prefusion Rsv F Vaccine Derived from Structural Analysis of the Fusion Mechanism. Nat. Commun..

[16] Joyce M.G., Zhang B.S., Ou L., Chen M., Chuang G.Y., Druz A., Kong W.P., Lai Y.T., Rundlet E.J., Tsybovsky Y. (2016). Iterative Structure-Based Improvement of a Fusion-Glycoprotein Vaccine against Rsv. Nat. Struct. Mol. Biol..

[17] Alandijany T.A., Qashqari F.S. (2025). Evaluating the Efficacy, Safety, and Immunogenicity of Fda-Approved Rsv Vaccines: A Systematic Review of Arexvy, Abrysvo, and Mresvia. Front. Immunol..

[18] Hause A.M., Moro P.L., Baggs J., Zhang B.C., Marquez P., Melgar M., Britton A., Stroud E., Myers T.R., Rakickas J. (2024). Early Safety Findings among Persons Aged ≥60 Years Who Received a Respiratory Syncytial Virus Vaccine-United States, May 3, 2023-April 14, 2024. Mmwr-Morb. Mortal. Wkly. Rep..

[19] Walsh E.E., Marc G.P., Zareba A.M., Falsey A.R., Jiang Q., Patton M., Polack F.P., Llapur C., Doreski P.A., Ilangovan K. (2023). Efficacy and Safety of a Bivalent Rsv Prefusion F Vaccine in Older Adults. N. Engl. J. Med..

[20] Mahase E. (2024). Fda Pauses All Infant Rsv Vaccine Trials after Rise in Severe Illnesses. BMJ.

[21] Kampmann B., Madhi S.A., Munjal I., Simoes E.A.F., Pahud B.A., Llapur C., Baker J., Marc G.P., Radley D., Shittu E. (2023). Bivalent Prefusion F Vaccine in Pregnancy to Prevent Rsv Illness in Infants. N. Engl. J. Med..

[22] Altenburg A.F., Kreijtz J.H.C.M., de Vries R.D., Song F., Fux R., Rimmelzwaan G.F., Sutter G., Volz A. (2014). Modified Vaccinia Virus Ankara (Mva) as Production Platform for Vaccines against Influenza and Other Viral Respiratory Diseases. Viruses.

[23] Volz A., Sutter G., Kielian M., Mettenleiter T.C., Roossinck M.J. (2017). Modified Vaccinia Virus Ankara: History, Value in Basic Research, and Current Perspectives for Vaccine Development. Advances in Virus Research.

[24] Orlova O.V., Glazkova D.V., Bogoslovskaya E.V., Shipulin G.A., Yudin S.M. (2022). Development of Modified Vaccinia Virus Ankara-Based Vaccines: Advantages and Applications. Vaccines.

[25] Wyatt L.S., Whitehead S.S., Venanzi K.A., Murphy B.R., Moss B. (1999). Priming and Boosting Immunity to Respiratory Syncytial Virus by Recombinant Replication-Defective Vaccinia Virus Mva. Vaccine.

[26] de Waal L., Wyatt L.S., Yüksel S., van Amerongen G., Moss B., Niesters H.G.M., Osterhaus A., de Swart R.L. (2004). Vaccination of Infant Macaques with a Recombinant Modified Vaccinia Virus Ankara Expressing the Respiratory Syncytial Virus F and G Genes Does Not Predispose for Immunopathology. Vaccine.

[27] Olszewska W., Suezer Y., Sutter G., Openshaw P.J.M. (2004). Protective and Disease-Enhancing Immune Responses Induced by Recombinant Modified Vaccinia Ankara (Mva) Expressing Respiratory Syncytial Virus Proteins. Vaccine.

[28] Endt K., Wollmann Y., Haug J., Bernig C., Feigl M., Heiseke A., Kalla M., Hochrein H., Suter M., Chaplin P. (2022). A Recombinant Mva-Based Rsv Vaccine Induces T-Cell and Antibody Responses That Cooperate in the Protection against Rsv Infection. Front. Immunol..

[29] Jordan E., Jenkins V., Silbernagl G., Chávez M.P.V., Schmidt D., Schnorfeil F., Schultz S., Chen L.D.Y., Salgado F., Jacquet J.M. (2024). A Multivalent Rsv Vaccine Based on the Modified Vaccinia Ankara Vector Shows Moderate Protection against Disease Caused by Rsv in Older Adults in a Phase 3 Clinical Study. Vaccine.

[30] Rhee C.H., Her M., Jeong W. (2022). Modified Vaccinia Virus Ankara as a Potential Biosafety Level 2 Surrogate for African Swine Fever Virus in Disinfectant Efficacy Tests. Pathogens.

[31] Wyatt L.S., Earl P.L., Moss B. (2017). Generation of Recombinant Vaccinia Viruses. Curr. Protoc. Protein Sci..

[32] Deng L., Cao H., Li G., Zhou K., Fu Z., Zhong J., Wang Z., Yang X. (2025). Progress on Respiratory Syncytial Virus Vaccine Development and Evaluation Methods. Vaccines.

[33] Bouzya B., Rouxel R.N., Sacconnay L., Mascolo R., Nols L., Quique S., François L., Atas A., Warter L., Dezutter N. (2023). Immunogenicity of an As01-Adjuvanted Respiratory Syncytial Virus Prefusion F (Rsvpref3) Vaccine in Animal Models. npj Vaccines.

[34] Schaerlaekens S., Jacobs L., Stobbelaar K., Cos P., Delputte P. (2024). All Eyes on the Prefusion-Stabilized F Construct, but Are We Missing the Potential of Alternative Targets for Respiratory Syncytial Virus Vaccine Design?. Vaccines.

[35] Beicht J., Kubinski M., Zdora I., Puff C., Biermann J., Gerlach T., Baumgärtner W., Sutter G., Osterhaus A., Prajeeth C.K. (2023). Induction of Humoral and Cell-Mediated Immunity to the Ns1 Protein of Tbev with Recombinant Influenza Virus and Mva Affords Partial Protection against Lethal Tbev Infection in Mice. Front. Immunol..

[36] Om K., Paquin-Proulx D., Montero M., Peachman K., Shen X.Y., Wieczorek L., Beck Z., Weiner J.A., Kim D., Li Y.F. (2020). Adjuvanted Hiv-1 Vaccine Promotes Antibody-Dependent Phagocytic Responses and Protects against Heterologous Shiv Challenge. PLoS Pathog..

[37] Pérez P., Martín-Acebes M.A., Poderoso T., Lázaro-Frías A., Saiz J.C., Sorzano C.O.S., Esteban M., García-Arriaza J. (2021). The Combined Vaccination Protocol of DNA/Mva Expressing Zika Virus Structural Proteins as Efficient Inducer of T and B Cell Immune Responses. Emerg. Microbes Infect..

[38] Marcos-Villar L., Perdiguero B., López-Bravo M., Zamora C., Sin L., Alvarez E., Sorzano C.O.S., Sánchez-Cordón P.J., Casasnovas J.M., Astorgano D. (2024). Heterologous Mrna/Mva Delivering Trimeric-Rbd as Effective Vaccination Regimen against SARS-CoV-2: Covarna Consortium. Emerg. Microbes Infect..

[39] McLellan J.S. (2015). Neutralizing Epitopes on the Respiratory Syncytial Virus Fusion Glycoprotein. Curr. Opin. Virol..

[40] Saeland E., van der Fits L., Bolder R., der Meer M.H.-V., Drijver J., van Polanen Y., Vaneman C., Tettero L., Serroyen J., Schuitemaker H. (2022). Immunogenicity and Protective Efficacy of Adenoviral and Subunit Rsv Vaccines Based on Stabilized Prefusion F Protein in Pre-Clinical Models. Vaccine.

[41] Ngwuta J.O., Chen M., Modjarrad K., Joyce M.G., Kanekiyo M., Kumar A., Yassine H.M., Moin S.M., Killikelly A.M., Chuang G.Y. (2015). Prefusion F-Specific Antibodies Determine the Magnitude of Rsv Neutralizing Activity in Human Sera. Sci. Transl. Med..

[42] van Erp E.A., Luytjes W., Ferwerda G., van Kasteren P.B. (2019). Fc-Mediated Antibody Effector Functions during Respiratory Syncytial Virus Infection and Disease. Front. Immunol..

[43] Zheng Y., Bian L.J., Zhao H.T., Liu Y.L., Lu J.C., Liu D.W., Zhang K., Song Y.S., Luo Y.S., Jiang C.L. (2020). Respiratory Syncytial Virus F Subunit Vaccine with As02 Adjuvant Elicits Balanced, Robust Humoral and Cellular Immunity in Balb/C Mice. Front. Immunol..

[44] Bian L.J., Zheng Y., Guo X.H., Li D.D., Zhou J.Y., Jing L.Y., Chen Y., Lu J.C., Zhang K., Jiang C.L. (2022). Intramuscular Inoculation of As02-Adjuvanted Respiratory Syncytial Virus (Rsv) F Subunit Vaccine Shows Better Efficiency and Safety Than Subcutaneous Inoculation in Balb/C Mice. Front. Immunol..

[45] Falsey A.R., Walsh E.E., Scott D.A., Gurtman A., Zareba A., Jansen K.U., Gruber W.C., Dormitzer P.R., Swanson K.A., Jiang Q. (2022). Phase 1/2 Randomized Study of the Immunogenicity, Safety, and Tolerability of a Respiratory Syncytial Virus Prefusion F Vaccine in Adults with Concomitant Inactivated Influenza Vaccine. J. Infect. Dis..

[46] Chai P., Shi Y., Yu J., Liu X., Yang M., Li D., Li K., Li S., Kong X., Zhang Q. (2025). An Oral Vaccine Based on the Ad5 Vector with a Double-Stranded Rna Adjuvant Protects Mice against Respiratory Syncytial Virus. Int. Immunopharmacol..

[47] Miao J., Li X.J., Li Y.W., Mao L.J., Suo W.K., Lan J.M. (2025). Single-Dose Intranasal Immunization with Chad68-Vectored Prefusion F Vaccines Confers Sustained Protection against Respiratory Syncytial Virus in Murine Models. Vaccines.

[48] Flanagan K.L., van Crevel R., Curtis N., Shann F., Levy O. (2013). Heterologous (“Nonspecific”) and Sex-Differential Effects of Vaccines: Epidemiology, Clinical Trials, and Emerging Immunologic Mechanisms. Clin. Infect. Dis..

[49] Blok B.A., Jensen K.J., Aaby P., Fomsgaard A., van Crevel R., Benn C.S., Netea M.G. (2019). Opposite Effects of Vaccinia and Modified Vaccinia Ankara on Trained Immunity. Eur. J. Clin. Microbiol. Infect. Dis..

[50] Weiss K.A., Christiaansen A.F., Fulton R.B., Meyerholz D.K., Varga S.M. (2011). Multiple Cd4+ T Cell Subsets Produce Immunomodulatory Il-10 during Respiratory Syncytial Virus Infection. J. Immunol..

[51] Sun J., Cardani A., Sharma A.K., Laubach V.E., Jack R.S., Müller W., Braciale T.J. (2011). Autocrine Regulation of Pulmonary Inflammation by Effector T-Cell Derived Il-10 during Infection with Respiratory Syncytial Virus. PLoS Pathog..

[52] Loebbermann J., Schnoeller C., Thornton H., Durant L., Sweeney N.P., Schuijs M., O’Garra A., Johansson C., Openshaw P.J. (2012). Il-10 Regulates Viral Lung Immunopathology during Acute Respiratory Syncytial Virus Infection in Mice. PLoS ONE.

[53] Sacco R.E., Durbin R.K., Durbin J.E. (2015). Animal Models of Respiratory Syncytial Virus Infection and Disease. Curr. Opin. Virol..

[54] Taylor G. (2017). Animal Models of Respiratory Syncytial Virus Infection. Vaccine.

