# Supplementary material for: Immunogenicity of a Recombinant MVA Vector Vaccine Expressing the Prefusion RSV F Protein in Balb/c Mice
Source: Vaccines (Basel). 2026 Mar 31;14(4):317. doi: 10.3390/vaccines14040317 (PMC13120400; doi:10.3390/vaccines14040317)
Supplement: Supplementary file 1 [file vaccines-14-00317-s001.zip › vaccines-4183154-supplementary.pdf]

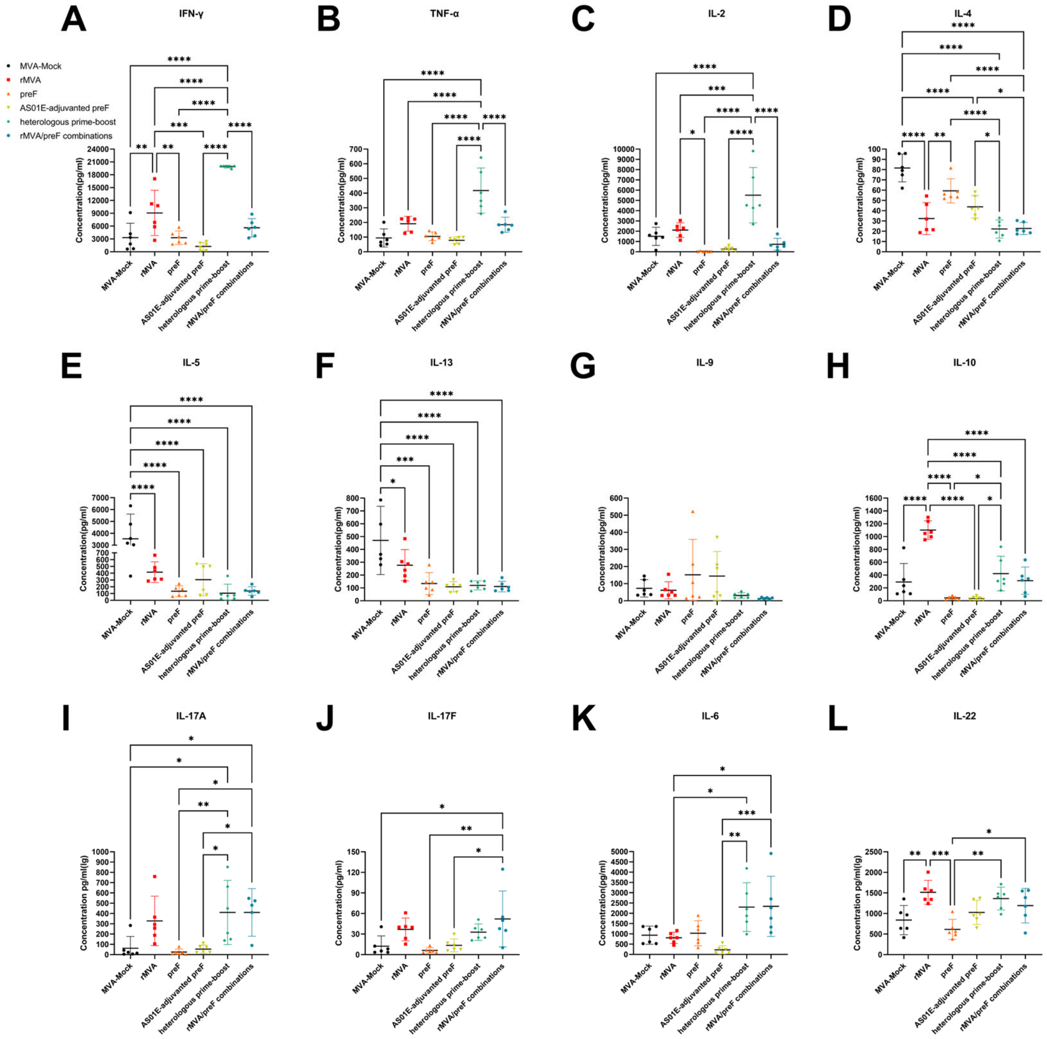


**Supplementary Figure S1.** Cytokine secretion profiles and intergroup differences following vaccination in mice. Mouse splenocytes were stimulated for 48h with an RSV A F protein peptide pool. Culture supernatants were collected and analyzed by LEGENDplex™ bead-based immunoassay for the following cytokines: IFN-γ (A), TNF-α (B), IL-2 (C), IL-4 (D), IL-5 (E), IL-13 (F), IL-9 (G), IL-10 (H), IL-17A (I), IL-17F (J), IL-6 (K), and IL-22 (L). Horizontal lines represent the group mean with 95% confidence intervals. **P*<0.05, ***P*<0.01, ****P*<0.001, *****P*<0.0001.
